# Supplementary material for: Phytochemical Profiling of Sambucus nigra L. Flower and Leaf Extracts and Their Antimicrobial Potential against Almond Tree Pathogens
Source: Int J Mol Sci. 2023 Jan 6;24(2):1154. doi: 10.3390/ijms24021154 (PMC9866908; doi:10.3390/ijms24021154)
Supplement: Supplementary file 1 [file ijms-24-01154-s001.zip › ijms-2114329-supplementary.pdf]

Phytochemical Profiling of *Sambucus nigra* L. Flower and Leaf Extracts and their Antimicrobial Potential against Almond Tree Pathogens

E. Sánchez-Hernández, J. Balduque-Gil, V. González-García, J.J. Barriuso-Vargas, J. Casanova-Gascón, J. Martín-Gil, and P. Martín-Ramos

SUPPLEMENTARY MATERIAL

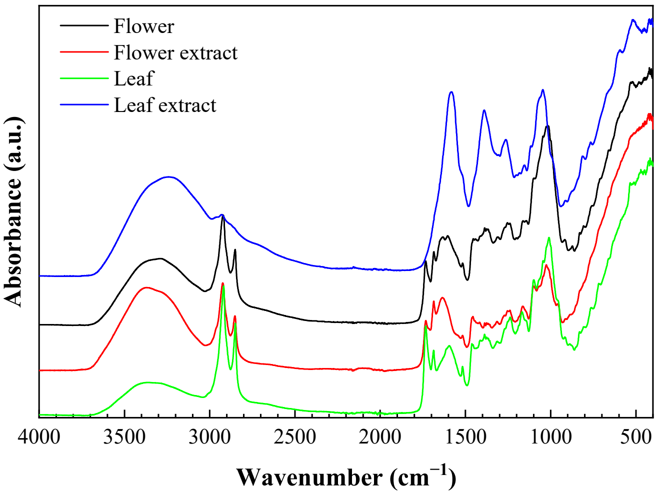

Figure S1. Infrared spectra of *S. nigra* plant organs and their aqueous ammonia extracts.

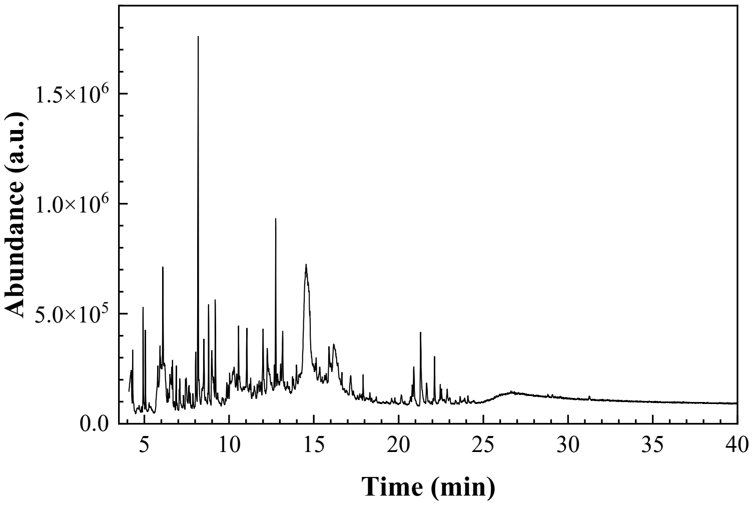

Figure S2. GC–MS chromatogram of *S. nigra* flower aqueous ammonia extract.

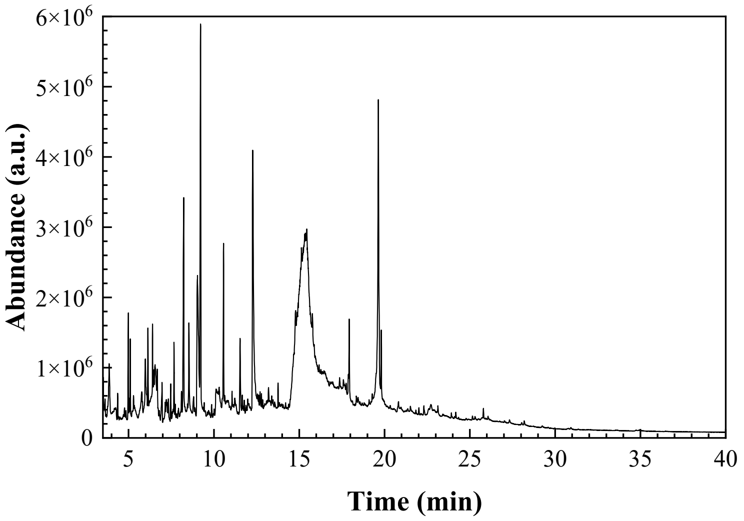

Figure S3. GC–MS chromatogram of *S. nigra* leaf aqueous ammonia extract.

Table S1. Main phytoconstituents identified in *S. nigra* flower aqueous ammonia extract.

RT (min)	Area (%)	Assignment
4.2292	0.9388	2-Furanmethanol
4.3123	0.5693	2-Propenamide
4.9296	1.2022	Butyrolactone
5.0602	0.8573	2-Cyclopenten-1-one, 2-hydroxy-
5.7961	1.5700	D-Arabinitol
5.9267	2.2750	Glycerin
6.0039	0.7626	4-Hydroxy-3-[[1,3-dihydroxy-2-propoxy]methyl]-1H-pyrazole-5-carboxamide
6.0929	3.5196	1-Methyl-2-piperidinemethanol
6.1938	1.9681	Glycerin
6.3837	0.3361	1-Pentyn-3-amine, 3-methyl-
6.4609	0.1947	2-Hydroxy-1-(1'-pyrrolidiyl)-1-buten-3-one
6.5143	1.0918	2,3-Anhydro-d-galactosan
6.6271	0.7592	Guanazine
6.6686	0.6778	L-Norleucine, methyl ester
6.8942	0.6294	2,5-Dimethyl-4-hydroxy-3(2H)-furanone
7.3097	0.2653	dl-Alanyl-l-leucine
7.4343	0.3470	Butanoic acid, 3-amino-
7.4699	0.5982	1,5,7-Octatrien-3-ol, 3,7-dimethyl-
7.6124	0.2838	Formamide, N,N-diethyl-
7.642	0.3292	6-Azabicyclo[3.2.1]octane
7.7073	0.2328	Pyrimidine-4,6-diol, 5-methyl-
8.0337	0.7650	Ethanamine, N-ethyl-N-nitroso-
8.1821	5.0580	3,5-Dihydroxy-6-methyl-2,3-dihydropyran-4-one
8.2296	0.6064	Benzene, 1-ethenyl-4-methoxy-
8.4077	0.4182	2(3H)-Furanone, dihydro-4-hydroxy-
8.7935	1.4277	3,7-Octadiene-2,6-diol, 2,6-dimethyl-
8.9775	1.6292	Catechol
9.1911	1.6099	Benzofuran, 2,3-dihydro-
9.8678	0.4464	1,2-Benzenediol, 3-methoxy-
9.9568	0.2642	Dicyclobutylidene oxide
10.034	0.7610	Pyrazine, 2-ethyl-5-methyl-
10.1467	0.4641	Hydroquinone
10.2239	0.8864	Hydroquinone
10.301	1.5422	4-Methyl-2-oxo-(1H)-pyrimidine
10.4553	0.9764	Pseudopelletierine
10.5563	1.1591	2-Methoxy-4-vinylphenol
10.6215	0.8577	1-Deoxy-d-arabitol

11.0548	1.6652	Geranic acid
11.221	0.4496	2-Amino-4-methyl-5-nitropyridine
11.2744	0.3925	Ethyl di-N-butylamine
11.4881	0.5765	4-Pentenoic acid, 2-(2-oxopropyl)-
11.6662	0.2938	1,4-Heptadiene, 3-methyl-
11.8798	0.5271	6-Hydroxymethyl-5-methyl-bicyclo[3.1.0]hexan-2-one
12.0045	1.9841	Benzoic acid, 4-methoxy-
12.2597	1.5667	Naphthalene-D8
12.3487	0.4307	Benzoic amide, 2,3-dihydroxy-
12.3784	0.7837	2,5-Dimethylanisole
12.6752	0.5655	3-Dipropylamino-1,2-propanediol
12.7583	2.2805	N,N-Diamylmethylamine
12.8592	0.5214	Meprobamate
12.8948	0.4273	Galacto-heptulose
13.0432	1.0174	Thiazole, 2-amino-5-methyl-
13.1678	1.6634	2-Hydroxy-1-(1'-pyrrolidiyl)-1-buten-3-one
13.4468	0.5002	Benzoic acid, 3-ethoxy-
13.8029	0.5031	2-Methylpyrazine-5-carboxylic acid
13.9809	1.0007	Dimethylmalonic acid, ethyl isohexyl ester
14.1174	0.5454	N-Acrylonitrylaziridine
14.1768	0.3354	1H-Imidazole-4-methanol, 5-methyl-
14.5507	10.5095	Octyl 2-methylpropanoate or Caprylyl isobutyrate
14.6101	2.1222	Heptane, 4-methyl-
14.6694	3.9431	Propylmalonic acid or Propanedioic acid, propyl-
14.7703	3.0347	Cyclopentanone, 2-cyclopentylidene-
15.0849	0.7310	4-Methylquinolinimide
15.1443	0.8503	6-Methoxypiperidin-2-one
15.3461	1.2235	2-Propenoic acid, 3-(4-methoxyphenyl)-
15.6428	0.6820	d-Talonic acid lactone
15.7378	0.4912	4-((1E)-3-Hydroxy-1-propenyl)-2-methoxyphenol
15.898	1.5142	1-Octanone, 1-(2-furanyl)-
16.0108	0.4612	2-Propen-1-amine, N,N-di-2-propenyl-
16.0227	0.4196	2-Propen-1-amine, N,N-di-2-propenyl-
16.1711	1.5306	Adenine
16.2007	1.2043	Adenine
16.2779	1.4258	Adenine
16.6578	0.2754	6-Cyano-5-methyl-1,3-diazaadamantan-6-ol
17.1801	0.7046	n-Hexadecanoic acid
17.9101	0.3332	n-Hexadecanoic acid
20.1655	0.3501	Heptanal
20.7116	0.2348	4-n-Pentylthiane, S,S-dioxide
20.8065	0.4226	1,3,3-Trimethyl-2-hydroxymethyl-3,3-dimethyl-4-(3-methylbut-2-enyl)-cyclohexene
20.9015	1.0163	Terpinen-4-ol
21.3051	2.1548	Heptane, 2,3-epoxy-
21.6612	0.6287	3,7-Nonadien-2-ol, 4,8-dimethyl-
22.4684	0.2667	Neopentylidenecyclohexane
22.5396	0.2827	2,6,10-Dodecatrien-1-ol, 3,7,11-trimethyl-

The appearance of several peaks for the same compound (e.g., for hydroquinone; 2-propen-1-amine, N,N-di-2-propenyl-; adenine; and n-hexadecanoic acid) is tentatively ascribed to matrix-induced retention shifts.

Table S2. Main phytoconstituents identified in *S. nigra* leaf aqueous ammonia extract.

RT (min)	Area (%)	Assignment
4.1730	0.2049	2-Furanmethanol
4.9862	0.5443	Butyrolactone
5.0990	0.3930	2-Cyclopenten-1-one, 2-hydroxy-
5.2948	0.2855	2(5H)-Furanone, 5-methyl-
5.3423	0.3491	1-Pentene, 5-methoxy-
5.7756	0.7217	Phenol
5.9833	1.1652	1,6-Anhydro-2,4-dideoxy-.beta.-D-ribo-hexopyranose
6.1317	0.6314	Ethanone, 1-phenyl-2-(1-piperidinyl)-
6.2385	0.2476	Glycerin
6.4107	1.1096	2H-Pyran-2-one, 5,6-dihydro-
6.4878	0.4230	Glycerin
6.5531	1.1073	Glycerin
6.6896	0.5143	Ethyl diazoacetate
6.9626	0.3529	2,5-Dimethyl-4-hydroxy-3(2H)-furanone
7.2119	0.2162	2-Pyrrolidinone
7.4731	0.2197	L-Alanine, methyl ester
7.6689	0.4170	2(1H)-Pyridinone, 6-hydroxy-
8.1082	0.3637	2-Propanamine, N-methyl-N-nitroso-
8.2328	2.1018	4H-Pyran-4-one, 2,3-dihydro-3,5-dihydroxy-6-methyl-
8.5414	1.0787	1,3-Disilacyclobutane, 1,1,3,3-tetramethyl-
8.8145	0.3444	3-Aminocrotononitrile
9.0400	2.7968	Catechol
9.2240	3.4864	Benzofuran, 2,3-dihydro-
10.1380	0.5962	Hydroquinone
10.2449	0.2097	Hydroquinone
10.3102	0.7560	Hydroquinone
10.5654	1.4085	2-Methoxy-4-vinylphenol
10.6366	0.3042	N,N'-Dimethyl-5-pyrrolidinone-3-carboxamide
10.7197	0.2356	5-Aminohexanoic acid
10.8503	0.2434	Butanal, 3-hydroxy-
10.9512	0.2123	Propanoic acid, 2-(aminooxy)-
11.0699	0.3037	Phenol, 2,6-dimethoxy-
11.1352	0.2088	Propanoic acid, 2-(aminooxy)-
11.2301	0.5883	1H-Pyrrole, 1-methyl-
11.5388	0.8415	1,3-Benzenediol, 4-ethyl-
11.6693	0.2182	Benzenamine, 2,6-diethyl-
11.8830	0.2044	Pyrazolo[3,4-f]indazole-3,7(2H,6H)-dione, 4,8-dihydro-
11.9899	0.2583	1-Butyn-3-one, 1-(6,6-dimethyl-1,2-epoxycyclohexyl)-
12.0492	0.2420	4,4'-Bipyridine
12.2807	4.4879	2,1,3-Benzothiadiazole
12.5596	0.3329	Benzoic acid, 2,3-dihydroxy-
12.6249	0.2067	Isopropyl (2-oxo-1,3-benzothiazol-3(2H)-yl)acetate
12.7080	0.3549	4-Piperidinamine, N,1-dimethyl-
12.7911	0.2006	4-(2,6,6-Trimethylcyclohexa-1,3-dienyl)but-3-en-2-one
12.8623	0.2529	2-Butenoic acid, 3-methyl-, methyl ester
12.9810	0.2370	3-Pyridinecarboxylic acid, 2-nitro-, methyl ester
13.2066	0.3394	2-Hydroxy-1-(1'-pyrrolidiyl)-1-buten-3-one
13.2659	0.3661	Benzoic acid, 3-hydroxy-
13.3906	0.3116	1-(4-methylthiophenyl)-2-propanone
13.4677	0.2605	Benzoic acid, 3-hydroxy-
13.5627	0.3416	3-Oxabicyclo[4.1.0]heptane-7-carboxamide, 6-methyl-N-(1-naphthyl)-
13.7645	0.3250	4-Methyl-2,5-dimethoxybenzaldehyde
14.2037	0.3043	2-Amino-3-cyano-5-aldoximinopyrazine-1-oxide
14.7913	3.8924	trans-3-Penten-2-ol
14.8863	1.3678	Butanoic acid, pentyl ester
14.9397	0.9262	Butanoic acid, pentyl ester
15.1296	5.7230	Butanoic acid, pentyl ester

15.2305	1.8771	4(1H)-Pyrimidinone, 6-hydroxy-
15.3611	4.7153	.beta.-D-Glucopyranose, 1,6-anhydro-
15.4264	1.0311	4(1H)-Pyrimidinone, 6-hydroxy-
15.4501	6.9039	.beta.-D-Glucopyranose, 1,6-anhydro-
15.7766	1.8842	4-((1E)-3-Hydroxy-1-propenyl)-2-methoxyphenol
15.8834	0.7291	Nonane, 4-methyl-5-propyl-
15.9487	1.9985	2,3-dihydroxycyclohexanone
16.1683	1.0358	(1's,2's)-Nicotine-N'-oxide
16.2751	0.5919	Nonane, 4-methyl-5-propyl-
16.3582	0.7736	3,4-Altrosan
16.4532	0.5294	Octyl-.beta.-D-glucopyranoside
16.4947	1.1402	Octane, 3-ethyl-
16.8093	0.5061	Ether, 6-methylheptyl vinyl
16.9102	0.7114	Adenosine 3',5'-cyclic monophosphate
16.9755	0.7125	1-Ethyl-6-methyl-1,2,3,4-tetrahydropyrrolo[1,2-a]pyrazine
17.2426	0.5945	d-Talonic acid lactone
17.3672	0.6816	Inositol, 1-deoxy-
17.4385	0.3960	Inositol, 1-deoxy-
17.5928	0.7440	Scyllo-Inositol
17.6699	0.4089	Myo-Inositol
17.7530	0.5122	2-Acetonylcyclohexanone
17.8836	0.5071	.+/-.-trans-2-Cyclohexene-1,4-diol
17.9370	1.1550	n-Hexadecanoic acid
18.1210	0.5150	Tridecanoic acid
18.3525	0.3688	3-Phenylbicyclo(3.2.2)nona-3,6-dien-2-one
18.4475	0.4575	1H-Indole, 4-(3-methyl-2-butenyl)-
18.6967	0.3634	Z-8-Methyl-9-tetradecenoic acid
18.8095	0.2383	Oleic Acid
18.8748	0.2269	Octadec-9-enoic acid
19.1003	0.4215	1-Heptadecene
19.2784	0.5507	Cyclopentadecanone, 2-hydroxy-
19.6405	5.7605	Oleic Acid
19.8126	0.9029	Octadecanoic acid
19.9194	0.4611	6-Octadecenoic acid
20.3349	0.4364	cis-7,cis-11-Hexadecadien-1-yl acetate
20.9581	0.4000	E-8-Methyl-7-dodecen-1-ol acetate
21.3676	0.2783	Oleic Acid
21.5219	0.2545	Cyclopropaneoctanal, 2-octyl-
21.8424	0.2051	8-Hexadecenal, 14-methyl-, (Z)-
22.7209	0.4395	Prunasin
22.8871	0.2534	4-Butylbenzonitrile

The appearance of several peaks for the same compound (e.g., for glycerin, hydroquinone; propanoic acid, 2-(aminooxy)-; benzoic acid, 3-hydroxy-; butanoic acid, pentyl ester; and inositol, 1-deoxy-) is tentatively ascribed to matrix-induced retention shifts. In the case of β-D-glucopyranose, 1,6-anhydro-; and 4(1H)-pyrimidinone, 6-hydroxy-, it may result from the existence of isomers.

Table S3. Effectiveness of *S. nigra* flower and leaf extracts against pathogens reported in the literature.

Collection site	Extraction procedure	Microorganisms	Inhibition values	Ref.
Local market in Braga (Portugal)	Flower aqueous extract	*Pseudomonas aeruginosa* PAO1	MIC >33,000 $\mu g \cdot mL^{-1}$	[1]
		Klebsiella oxytoca ATCC 13182	MIC >33,000 $\mu g \cdot mL^{-1}$	
		K. pneumonia ATCC 11296	MIC >33,000 $\mu g \cdot mL^{-1}$	
		Staphylococcus aureus ATCC 25293	MIC = 8300 $\mu g \cdot mL^{-1}$	
		S. epidermidis ATCC 12228	MIC = 4100 $\mu g \cdot mL^{-1}$	
		Candida albicans SC 5314	MIC >33,000 $\mu g \cdot mL^{-1}$	
Northern Ireland	Flower water:ethane 96% (50:50) extract	MRSA	IZ = 17 mm	[2]
		P. aeroginosa	IZ = 9 mm	
		Staphylococcus sp	IZ = 5−14 mm	
		Bacillus cereus	IZ = 5−14 mm	
		Salmonella poona	IZ = 5−14 mm	
		P. aeroginosa	IZ = 5−14 mm	
Insol (Slovenia)	Flower SFE-CO_2 extract, 10 %	*Alternaria alternata*	PGI = −2.13 ± 2.09 %	[3]
		Epicoccum nigrum	PGI = 11.36 ± 8.53 %	
		Botrytis cinerea	PGI = 81.13 ± 3.68 %	
		Fusarium oxysporum	PGI = 18.66 ± 1.82 %	
		F. poae	PGI = 75.21 ± 2.70 %	
Lesser Poland Voivodeship (Poland)	Flower ethanol (80%), methanol (80%) or water extracts	*B. subtilis* subsp. *subtilis* DSM 10	IZ = n.a.−1.17 mm	[4]
		Bifidobacterium sp. DSM 20104	IZ = n.a.−0.22 mm	
		Clostridium sp. DSM 756	IZ = n.a.−0.11 mm	
		Escherichia coli DSM 4261	IZ = n.a.−1.44 mm	
		Micrococcus luteus DSM 20030	IZ = 0.17−1.33 mm	
		Proteus myxofaciens DSM 4482	IZ = 0.17−1.44 mm	
		P. putida DSM 291	IZ = 0.11−1.22 mm	
		Serratia marcescens DSM 1636	IZ = n.a.−0.94	
		Aspergillus niger CBS 10930	n.a.	
		Penicillium chrysogenum DSM 844	IZ = n.a.−0.17 mm	
		Saccharomyces cerevisiae DSM 1333	IZ = 0.11−0.94 mm	
Northern Ireland	Leaf water extract	MRSA	n.a.	[2]
		B. cereus	IZ = 6 mm	
		S. marcescens	IZ = 6 mm	
		E. coli 0157	IZ = 7 mm	

IZ = Inhibition Zone (mm); MIC = Minimum Inhibition Concentration; MRSA = methicillin-resistant *S. aureus*; n.a. = no activity; PGI = percentage growth inhibition; SFE-CO_2 = Supercritical fluid extraction using CO_2.

Table S4. Antifungal and anti-oomycete activities reported in the literature for other natural products against two of the phytopathogens studied in this work.

Pathogen	Natural Product	Effective Concentration / MIC (μg·mL^{-1})	Ref.
P. megasperma	*Ageratum houstonianum* EO	EC_{90} = 422.28	[5]
V. dahliae	*Sambucus nigra* flower extract	MIC = 1500	This work
	S. nigra leaf extract	MIC = 1500	
	Uncaria tomentosa bark extract	MIC = 500	[6]
	Origanum heracleoticum PE	MIC > 5×10^5	[7]
	Salvia officinalis PE	MIC > 5×10^5	
	Rosmarinus officinalis PE	MIC > 5×10^5	
	Propolis	MIC > 60,000	[8]
	Mentha piperita PE	MIC >1×10^5	[9]
	Thymus vulgaris PE	MIC >1×10^5	
	Lavandula angustifolia PE	MIC >1×10^5	
	Artemisia argyi PE	EC_{50} = 1227	[10]
	Haplophyllum tuberculatum PE	MIC > 1000	[11]
	Chrysanthemum coronarium PE	MIC = 1000	
	O. vulgare EO	MIC = 500	[12]
	Allium sativum PE	MIC > 5000	[13]
	Allium sp. PE	MIC > 5000	
	Atropa belladona PE	MIC > 5000	
	Azadirachta indica PE	MIC > 5000	
	Castanea sativa PE	MIC > 5000	
	Citrus aurantium PE	MIC > 5000	
	Citrus sp. PE	MIC = 5000	
	Inula viscosa PE	MIC > 5000	
	Juniperus communis PE	MIC > 5000	
	Olea europaea cv. Frantoio PE	MIC > 5000	
	Olea europea cv. Lechín PE	MIC > 5000	
	Papaver rhoeas PE	MIC > 5000	
	Pistacia lentiscus PE	MIC > 5000	
	Salvia officinalis PE	MIC > 5000	
	Sambucus nigra PE	MIC > 5000	
	Thymus sp. PE	MIC ≥ 5000	
	Cymbopogon sp. EO	MIC > 5000	
	Eucaliptus sp. EO	MIC > 5000	
	Illicium verum EO	MIC > 5000	

Laurus nobilis EO	MIC > 5000
Melaleuca alternifolia EO	MIC > 5000
Melaleuca cajeputi EO	MIC = 5000
Mentha sp. EO	MIC > 5000
Mirtus communis EO	MIC > 5000
Origanum vulgare EO	MIC > 5000
Pinus sp. EO	MIC > 5000
Rosmarinus officinalis EO	MIC > 5000
Salvia officinalis EO	MIC > 5000
Satureja sp. EO	MIC = 5000
Thymus sp. EO	MIC ≥ 5000
Verbena officinalis EO	MIC = 5000

EO = essential oil; PE = plant extract

Table S5. Antimicrobial and oomyceticidal activity reported in the literature for other natural products rich in the main phytochemicals found in the flower extract of *S. nigra*, and for pure compounds.

Phytochemical	Plant	Content (%)	Microorganism	Inhibition values	Ref.
Octyl isobutyrate	*Sambucus nigra* flower	10.5	*Diaporthe amygdali*	MIC = 1000 μg·mL^{-1}	This work
			Phytophthora megasperma	MIC = 375 μg·mL^{-1}	
			Verticillium dahliae	MIC = 1500 μg·mL^{-1}	
	Malabaila aurea aerial parts EO	40	*Staphylococcus aureus*	MIC = 4.5 μg·mL^{-1}	[14]
			Candida albicans	MIC = 1.125 μg·mL^{-1}	
	Heracleum persicum EO	17.82	*Listeria monocytogenes*	MIC = 2500 μg·mL^{-1}	[15]
			L. monocytogenes	MIC = 5200 μg·mL^{-1}	[16]
			Escherichia coli	MIC = 5000 μg·mL^{-1}	
	Elaeosticta glaucescens roots EO	2.8	*E. coli*	MIC = 1000 μg·mL^{-1}	[17]
			Pseudomonas aeruginosa	MIC = 1000 μg·mL^{-1}	
			S. aureus	MIC = 1000 μg·mL^{-1}	
			Bacillus subtilis	MIC = 1000 μg·mL^{-1}	
	Caucalis patycarpos roots EO	8.5	*E. coli*	MIC = 500 μg·mL^{-1}	
			P. aeruginosa	MIC = 1000 μg·mL^{-1}	
			S. aureus	MIC = 500 μg·mL^{-1}	
			Bacillus subtilis	MIC = 500 μg·mL^{-1}	
	Eryngium caucasicum roots EO	4.0	*E. coli*	MIC = 500 μg·mL^{-1}	
			P. aeruginosa	MIC >1000 μg·mL^{-1}	
			S. aureus	MIC = 500 μg·mL^{-1}	
			B. subtilis	MIC = 500 μg·mL^{-1}	
	Heracleum sphondylium subsp. *ternatum* EO	24.6	*B. cereus*	MIC = 125 μg·mL^{-1}	[18]
			Enterobacter aerogenes	MIC = 500 μg·mL^{-1}	
			E. coli	MIC = 500 μg·mL^{-1}	
			Klebsiella pneumoniae	MIC = 125 μg·mL^{-1}	
			L. monocytogenes	MIC = 125 μg·mL^{-1}	
			P. syringae pv. *phaseolicola*	MIC = 125 μg·mL^{-1}	
			P. syringae pv. *syringae*	MIC = 31.25 μg·mL^{-1}	
			P. syringae pv. *tomato*	MIS = 500 μg·mL^{-1}	
			Proteus vulgaris	MIC = 500 μg·mL^{-1}	
			P. aeruginosa	MIC = 1000 μg·mL^{-1}	
			Salmonella typhimurium	MIC = 500 μg·mL^{-1}	
			S. aureus	MIC = 500 μg·mL^{-1}	
			S. epidermis	MIC = 250 μg·mL^{-1}	

			Xanthomonas campestris pv. *phaseoli*	MIC = 31.25 µg·mL^{-1}	
			X. campestris	MIC = 31.25 µg·mL^{-1}	
			Yersinia entocolitica	MIC = 125 µg·mL^{-1}	
			C. albicans	MIC = 500 µg·mL^{-1}	
4H-pyran derivatives	*Sambucus nigra* flower	5	*D. amygdali*	MIC = 1000 µg·mL^{-1}	This work
			P. megasperma	MIC = 375 µg·mL^{-1}	
			V. dahliae	MIC = 1500 µg·mL^{-1}	
	Punica granatum var. *nana* fruits	9.7	*Diplodia seriata* Y-084-01-01a	MIC > 1500 µg·mL^{-1}	[19]
			Erwinia amylovora NCPPB 595	MIC = 1500 µg·mL^{-1}	
			E. vitivora CCUG 21976	MIC = 1500 µg·mL^{-1}	
	Eucalyptus camaldulensis leaves or bark	0.05-3.05	*E. coli* ATCC25922	MIC = 12.5-25 µg·mL^{-1}	[20]
			P. aeruginosa ATCC27853	MIC = 6.25−25 µg·mL^{-1}	
			S. aureus ATCC24213	MIC = 0.391−25 µg·mL^{-1}	
			B. subtilis	MIC = 0.391−12.5 µg·mL^{-1}	
	Chemical synthesis of 4H-pyran derivatives		*S. aureus*	MIC = 32−512 µg·mL^{-1}	[21]
			S. pyrogenes	MIC = 64−512 µg·mL^{-1}	
			E. coli	MIC ≤ 512 µg·mL^{-1}	
			P. aeruginosa	MIC ≤ 512 µg·mL^{-1}	
	Chemical synthesis of 4H-pyran derivatives		*B. cereus* ATCC 14579	IZ = 4.50−29 mm	[22]
			B. subtilis ATCC 6633	IZ = 4.50−28 mm	
			Enteroccocus faecalis ATCC 29122	IZ =5.50−24.50 mm	
			S. aureus ATCC 25923	IZ = 5.75-27.50 mm	
			S. epidermidis ATCC 14990	IZ = 4.50−29.75 mm	
			E. coli ATCC 25966	IZ = 1.5−11mm	
			K. pneumonia ATCC 700603	IZ = 1.5−12 mm	
			P. aeruginosa ATCC 27853	IZ = 2.5−14-5 mm	
			S. enterica ATCC 43972	IZ = 1.5−14.5 mm	
Piperidine alkaloid derivatives	*Sambucus nigra* flower	3.5	*D. amygdali*	MIC = 1000 µg·mL^{-1}	This work
			P. megasperma	MIC = 375 µg·mL^{-1}	
			V. dahliae	MIC = 1500 µg·mL^{-1}	
	Mentha x rotundifolia leaves EO	2.66-3	*E. coli*	IZ = 33−34 mm	[23]
			S. typhimurium	IZ = 30−31 mm	
			S. aureus	IZ = 24 mm	
			B. cereus	IZ = 22−23 mm	
			A. niger	IZ = 22−23 mm	
			C. albicans	IZ = 20−21 mm	
			S. aureus ATCC 25923	MIC = 10.72 µg·mL^{-1}	[24]

	Chemical synthesis of piperidine derivatives	*B. cereus* ATCC 10987	MIC = 10.75 μg·mL^{-1}	
		S. enterica ATCC 43845	MIC = 43 μg·mL^{-1}	
		E. faecalis ATCC 29212	MIC = 5.37 μg·mL^{-1}	
		P. aeruginosa ATCC 27853	MIC = 5.37 μg·mL^{-1}	
		C. albicans ATCC 10231	MIC = 5.37 μg·mL^{-1}	
		C. glabrata ATCC 2001	MIC = 5.37 μg·mL^{-1}	
	Chemical synthesis of piperidine derivatives	*E. coli*	IZ = 6-12 mm at 10,000 g·mL^{-1}	[25]
		S. aureus	IZ = 17-24 mm at 10,000 g·mL^{-1}	

EO = essential oil; MIC = Minimum Inhibition Concentration; IZ = Inhibition Zone; n.a.= no activity; n.e. = not specified.

References

1. Ferreira-Santos, P.; Badim, H.; Salvador, Â.C.; Silvestre, A.J.D.; Santos, S.A.O.; Rocha, S.M.; Sousa, A.M.; Pereira, M.O.; Wilson, C.P.; Rocha, C.M.R.; Teixeira, J.A.; Botelho, C.M. Chemical Characterization of Sambucus nigra L. Flowers Aqueous Extract and Its Biological Implications. *Biomolecules* **2021**, *11*, doi:10.3390/biom11081222.
2. Caroline, H.; Graham, M.; David, N.; Linda, M.B.; Colin, E.G.; Paul, J.R.; Anne, L.; John, E.M.; Juluri, R.R. Antibacterial activity of elder (*Sambucus nigra* L.) flower or berry against hospital pathogens. *Journal of Medicinal Plants Research* **2010**, *4*, 1805-1809.
3. Schoss, K.; Kočevar Glavač, N.; Dolenc Koce, J.; Anžlovar, S. Supercritical CO2 Plant Extracts Show Antifungal Activities against Crop-Borne Fungi. *Molecules* **2022**, *27*, doi:10.3390/molecules27031132.
4. Cioch, M.; Satora, P.; Skotniczny, M.; Semik-Szczurak, D.; Tarko, T. Characterisation of antimicrobial properties of extracts of selected medicinal plants. *Polish Journal of Microbiology* **2017**, *66*, 463-472, doi:10.5604/01.3001.0010.7002.
5. Ramadan, K.M.A.; Ali, M.K.; Georghiou, P.E. Natural fungitoxicants of essential oil from *Ageratum houstonianum* L. and its application in control the root-rot diseases. *J. Biol. Chem. Environ. Sci* **2012**, *7*, 437-453.
6. Sánchez-Hernández, E.; Martín-Ramos, P.; Martín-Gil, J.; Santiago-Aliste, A.; Hernández-Navarro, S.; Oliveira, R.; González-García, V. Bark extract of *Uncaria tomentosa* L. for the control of strawberry phytopathogens. *Horticulturae* **2022**, *8*, 672, doi:10.3390/horticulturae8080672.
7. Salamone, A.; Zizzo, G.V.; Scarito, G. The antimicrobial activity of water extracts from Labiatae. *Acta Horticulturae* **2006**, 10.17660/ActaHortic.2006.723.67, 465-470, doi:10.17660/ActaHortic.2006.723.67.
8. Er, Y. *In vitro* and *in vivo* antimicrobial activity of propolis extracts against various plant pathogens. *Journal of Plant Diseases and Protection* **2021**, *128*, 693-701, doi:10.1007/s41348-021-00437-y.
9. Erdoğan, O.; Celik, A.; Zeybek, A. *In vitro* antifungal activity of mint, thyme, lavender extracts and essential oils on *Verticillium dahliae* Kleb. *Fresenius Environ. Bull.* **2016**, *25*, 4856-4862.
10. Wang, Y.; Li, J.; Chen, Q.; Zhou, J.; Xu, J.; Zhao, T.; Huang, B.; Miao, Y.; Liu, D. The role of antifungal activity of ethyl acetate extract from *Artemisia argyi* on *Verticillium dahliae*. *Journal of Applied Microbiology* **2021**, *132*, 1343-1356, doi:10.1111/jam.15298.
11. Abdelgaleil, S.A.M.; Saad, M.M.G.; Ariefta, N.R.; Shiono, Y. Antimicrobial and phytotoxic activities of secondary metabolites from *Haplophyllum tuberculatum* and *Chrysanthemum coronarium*. *South African Journal of Botany* **2020**, *128*, 35-41, doi:10.1016/j.sajb.2019.10.005.
12. Rus, C.F.; Alexa, E.; Sumalan, R.M.; Galuscan, A.; Dumitrache, A.; Imbrea, I.M.; Sarac, I.; Pag, A.; Pop, G. Antifungal activity and chemical composition of *origanum vulgare* L. essential oil. *Rev. Chim.* **2016**, *67*, 2287-2290.
13. Varo, A.; Mulero-Aparicio, A.; Adem, M.; Roca, L.F.; Raya-Ortega, M.C.; López-Escudero, F.J.; Trapero, A. Screening water extracts and essential oils from Mediterranean plants against *Verticillium dahliae* in olive. *Crop Protection* **2017**, *92*, 168-175, doi:10.1016/j.cropro.2016.10.018.
14. Tzakou, O.; Mylonas, P.; Hancianu, M.; Poiata, A. Composition and Antimicrobial Activity ofMalabaila aureaBoiss. Essential Oil. *Journal of Essential Oil Research* **2008**, *20*, 270-271, doi:10.1080/10412905.2008.9700009.
15. Ehsani, A.; Rezaeiyan, A.; Hashemi, M.; Aminzare, M.; Jannat, B.; Afshari, A. Antibacterial activity and sensory properties of *Heracleum persicum* essential oil, nisin, and *Lactobacillus acidophilus* against *Listeria monocytogenes* in cheese. *Veterinary World* **2019**, *12*, 90-96, doi:10.14202/vetworld.2019.90-96.
16. Rezayan, A.; Ehsani, A. Evaluation of the chemical compounds and antibacterial properties of the aerial parts of persian *Heracleum persicum* essence. *Journal of Babol University of Medical Sciences* **2015**, *17*, 26-32.
17. Hamedi, A.; Pasdaran, A.; Pasdaran, A. Antimicrobial activity and analysis of the essential oils of selected endemic edible Apiaceae plants root from Caspian Hyrcanian region (North of Iran). *Pharmaceutical Sciences* **2019**, *25*, 138-144, doi:10.15171/ps.2019.21.
18. İşcan, G.; Demirci, F.; Kürkçüoğlu, M.; Kıvanç, M.; Can Başer, K.H. The bioactive essential oil of *Heracleum sphondylium* L. subsp. *ternatum* (Velen.) Brummitt. *Zeitschrift für Naturforschung C* **2003**, *58*, 195-200, doi:10.1515/znc-2003-3-410.
19. Sánchez-Hernández, E.; Buzón-Durán, L.; Cuchí-Oterino, J.A.; Martín-Gil, J.; Lorenzo-Vidal, B.; Martín-Ramos, P. Dwarf pomegranate (*Punica granatum* L. var. *nana*): Source of 5-HMF and bioactive compounds with applications in the protection of woody crops. *Plants* **2022**, *11*, 550, doi:10.3390/plants11040550.
20. Alghamdi, A.I.; Ababutain, I.M. Phytochemical screening and antibacterial activity of *Eucalyptus camaldulensis*'s leaves and bark extracts. *Asian Journal of Scientific Research* **2019**, *12*, 202-210, doi:10.3923/ajsr.2019.202.210.

21. Nazari, P.; Bazi, A.; Ayatollahi, S.A.; Dolati, H.; Mahdavi, S.M.; Rafighdoost, L.; Amirmostofian, M. Synthesis and evaluation of the antimicrobial activity of spiro-4h-pyran derivatives on some Gram positive and Gram negative bacteria. *Iranian Journal of Pharmaceutical Research* **2017**, *16*, 943-952.
22. El-Sayed, N.N.E.; Zaki, M.E.A.; Al-Hussain, S.A.; Ben Bacha, A.; Berredjem, M.; Masand, V.H.; Almarhoon, Z.M.; Omar, H.S. Synthesis and evaluation of some new 4H-pyran derivatives as antioxidant, antibacterial and anti-HCT-116 cells of CRC, with molecular docking, antiproliferative, apoptotic and ADME investigations. *Pharmaceuticals* **2022**, *15*, 891, doi:10.3390/ph15070891.
23. Riahi, L.; Elferchichi, M.; Ghazghazi, H.; Jebali, J.; Ziadi, S.; Aouadhi, C.; Chograni, H.; Zaouali, Y.; Zoghlami, N.; Mliki, A. Phytochemistry, antioxidant and antimicrobial activities of the essential oils of *Mentha rotundifolia* L. in Tunisia. *Industrial Crops and Products* **2013**, *49*, 883-889, doi:10.1016/j.indcrop.2013.06.032.
24. Fyhrquist, P.; Virjamo, V.; Hiltunen, E.; Julkunen-Tiitto, R. Epidihydropinidine, the main piperidine alkaloid compound of Norway spruce (*Picea abies*) shows promising antibacterial and anti-*Candida* activity. *Fitoterapia* **2017**, *117*, 138-146, doi:10.1016/j.fitote.2017.01.011.
25. Shaikh, T.M.A.; Ammare, Y. Synthesis and evaluation of antimicrobial activities of new piperidine derivatives. *Biointerface Research in Applied Chemistry* **2020**, *10*, 7177-7186, doi:10.33263/briac106.71777186.
